# A prostate biopsy strategy based on a new clinical nomogram reduces the number of biopsy cores required in high-risk patients

**DOI:** 10.1186/1471-2490-14-8

**Published:** 2014-01-11

**Authors:** Yuan Huang, Gong Cheng, Bianjiang Liu, Pengfei Shao, Chao Qin, Jie Li, Lixin Hua, Changjun Yin

**Affiliations:** 1 State Key Laboratory of Reproductive Medicine, Department of Urology, First Affiliated Hospital of Nanjing Medical University, 300 Guangzhou Rd, Nanjing 210029, China

**Keywords:** Prostate cancer, Biopsy, Nomogram, Diagnosis

## Abstract

**Background:**

The nomograms used for prostate cancer risk assessment in Western countries are not directly applicable to Chinese males; consequently, we have developed a new model to evaluate the risk of them developing this disease.

**Methods:**

A total of 1104 patients who had undergone trans-rectal ultrasound (TRUS)-guided 12 + 1-core prostate biopsy were retrospectively evaluated in the first stage of the study. Age, prostate-specific antigen (PSA), the free/total PSA ratio (f/t), digital rectal examination (DRE) findings, the presence of a hypoechoic mass revealed using ultrasound, ultrasonic detection of microcalcifications, prostate volume (PV) and PSA density were considered as predictive factors. Multiple logistic regression analysis involving a backward elimination selection procedure was used to select independent predictors. We compared positive rates regarding 6-core and 12-core biopsy schemes at different risk levels. In the second stage of the study, 238 cases were evaluated using our nomogram. In higher risk patients, we employed a 6 + 1 core biopsy. Positive rates in the first and second stages of the study were compared.

**Results:**

Age, the baseline median natural logarithm of PSA (Ln[PSA]), Ln(PV), f/t, rate of abnormal DRE findings and rate of hypoechoic masses detected using TRUS were the factors that were finally submitted into our nomogram. A significantly greater area under the receiver-operating characteristic curve was obtained for the nomogram than for PSA level alone (0.853 vs. 0.761). A cancer probability cutoff value of 0.5 suggested no significant difference between the 6-core and 12-core biopsy schemes at higher risk levels. In the second stage of the study we verified that in patients with a cancer probability cutoff value >0.5, a 6 + 1-core biopsy could be used without a reduction in the positive detection rate, and significantly reducing the number of biopsy cores required.

**Conclusions:**

A nomogram based on data from Chinese males was developed to predict the positive detection rate, ratio of positive cores and Gleason score at each risk level. According to this nomogram, a reasonable biopsy strategy could be constituted to reduce the number of biopsy cores required in subjects at high risk.

## Background

Nowadays, prostate-specific antigen (PSA) is the most widely used biomarker for prostate cancer diagnosis. Nevertheless, the specificity of PSA in predicting prostate cancer is not satisfactory [1]. Some evolutional indexes, such as PSA velocity, PSA density (PSAD) and free/total PSA ratio (f/t), are used clinically; however, they are all provincial because of their dependence on PSA [2,3]. Nomograms, which are based on multiple independent risk factors for prostate cancer, have shown their superiority in detecting this cancer [4]. Although there are two well-known nomograms that have been used for incorporating known risk factors, namely in the Prostate Cancer Prevention Trial (PCPT) and in the European Randomized Study of Screening for Prostate Cancer (ERSPC), previous research has shown that they may not be directly applicable to Chinese males [5-7]. Therefore, it is necessary to develop a new prostate cancer risk assessment nomogram designed for the Chinese population.

The trans-rectal ultrasound (TRUS)-guided sextant biopsy method has been considered a standard method for obtaining prostatic tissues for histological evaluation since it was introduced by Hodge et al. [8] in 1989. Since then, surgeons have used additional cores to improve the accuracy of detection of prostate cancer [9-14]. Obviously, the extraction of additional cores can lead to excessive harm to the patient. According to our biopsy experience, as a result of the lack of PSA screening in most regions of China, a large number of positive cases of prostate cancer involving more than six positive cores have been diagnosed. In the present study, with the help of our nomogram we have attempted to identify cases in which we could reduce the number of biopsy cores required.

## Methods

### Ethics statement

This study was approved by the institutional review board of the First Affiliated Hospital of Nanjing Medical University. Written informed consent was obtained from all patients with regard to the storage of their information for the purpose of research. All research procedures were conducted in accordance with the Declaration of Helsinki.

### First stage

The first stage of this study included 1104 patients who had undergone a TRUS-guided prostate biopsy from July 2009 to September 2012 at the First Affiliated Hospital of Nanjing Medical University, China. A proportion of the patients had elevated PSA levels or abnormal digital rectal examination (DRE) findings during routine physical examination; the remaining patients had lower urinary tract symptoms with elevated PSA. Detailed patient information, including age, PSA, free (f)PSA and DRE findings, was recorded before biopsy. Every patient underwent calculation of prostate volume (PV) using an ellipsoid formula (PI/6*lateral*anteroposterior*superoinferior diameters) by means of ultrasonoscopy, and detailed observations were carried out regarding hypoechoic lesions and microcalcifications. PSAD was defined as the ratio of PSA to PV. The percentage of fPSA (the free/total PSA ratio [f/t]) was calculated by dividing the level of fPSA present by the total level of PSA.

The biopsy scheme included 13 cores; six mid cores were performed as a traditional 6-core biopsy, another six cores were performed parallel and lateral to the six mid cores, and the final core was directed at a hypoechoic lesion under ultrasound or performed at the apex of the prostate. The locations of positive cores were recorded in detail. We supposed that every patient received two biopsy schemes, namely systemic 12-core and traditional 6-core. After analysis of the locations of positive cores, the results from 6-core and 12-core biopsy were recorded.

Multiple logistic regression analysis with a backward elimination selection procedure was used to select independent predictors of prostate cancer in the model-building set. An equation for prostate cancer probability (PCP) was developed based on the final logistic regression model. On the basis of the value obtained from the PCP equation, we classified risk into five levels and evaluated the rate of positives, the Gleason score and the average number of positive cores at every risk level. Furthermore, we compared the differences in the positive rate between 6-core and 12-core biopsies at the various levels of risk and determined a rational biopsy strategy. With the help of our model, we found a PCP cutoff value to identify the patients in which there was no significant difference in the rate of positives between 6-core and 12-core biopsies. In these patients 6 + 1-core biopsy was considered more reasonable than 12 + 1-core biopsy.

### Second stage

In the second stage of the study, we prospectively investigated whether or not a reduction in the number of biopsy cores in high-risk patients would lead to a decrease in the rate of positive cases. A total of 238 patients were evaluated using our model and patients at high risk were recommended for the 6 + 1 biopsy scheme. We compared the positive core rate and the number of cores taken in the first and second stages of the study.

### Statistical analysis

Statistical analysis was performed using SPSS 18.0 software. Differences between data sets were analyzed using the t-test. Data were expressed as the mean ± standard deviation. The Chi-square test was used to compare categorical variables. A P value < 0.05 was considered as being statistically significant.

## Results

Among 1104 consecutive patients in the first stage of the study, 458 (41.49%) had positive biopsy results. Patient characteristics are detailed in Table 1. The mean ages of patients in the cancer and non-cancer groups in our study cohort were 70.8 ± 6.9 years and 67.4 ± 8.4 years, respectively (P < 0.001). The PSA level (P < 0.001), PV (P < 0.001), PSAD (P < 0.001), f/t (P < 0.001), and the rates of abnormal DRE (P < 0.001) findings and hypoechoic masses detected using TRUS (P < 0.001) differed significantly between the cancer and the non-cancer groups (Table 2). Because the PSA level and the PV had a non-normal distribution, these variables were transformed to the natural logarithm before univariate analysis. Univariate analysis indicated that age, Ln(PSA), Ln(PV), f/t, the rate of abnormal DRE and the rate of hypoechoic lesions on TRUS in patients with a positive initial biopsy were all significantly different from patients with a negative biopsy.

**Table 1:** Characteristics of the patient cohort in the first stage of the study

**Variables**	POS^*^		NEG^*^		**P**
	**N**	**(%)**	**N**	**(%)**	
Number of patients	458	(41.49)	646	(58.51)	
Age	70.8 ± 6.9		67.4 ± 8.4		<0.001
PSA	120.4 ± 15.0		13.7 ± 18.0		<0.001
PV	41.2 ± 24.4		48.2 ± 27.6		<0.001
PSAD	2.9 ± 13.5		0.3 ± 0.4		<0.001
f/t	0.12 ± 0.07		0.16 ± 0.10		<0.001
DRE findings					<0.001
neg^*^	234	(28.6)	585	(71.4)	
pos^*^	224	(78.6)	61	(21.4)	
Hypoechoic^#^					<0.001
neg^*^	307	(37.2)	518	(62.8)	
pos^*^	151	(54.1)	128	(45.9)	
Microcalcification^#^					0.592
neg*	319	(40.9)	460	(59.1)	
pos*	139	(42.8)	186	(57.2)	

*neg, negative; *pos, positive.

^#^Hypoechoic masses and microcalcifications were observed using ultrasound.

**Table 2:** Multivariate analysis of the predictors of prostate cancer

**Variables**	**OR**	**95% CI**		**P**
Age	1.056	1.034	1.078	<0.001
Ln(PSA)	2.218	1.552	3.169	<0.001
Ln(PV)	0.311	0.2	0.483	<0.001
PSAD	1.322	0.791	2.207	0.287
f/t	0.021	0.002	0.187	0.001
DRE findings^*^	5.276	3.578	7.78	<0.001
Hypoechoic^*^	1.562	1.095	2.229	0.014
Microcalcification^*^	1.045	0.748	1.46	0.799

*Reference category was negative.

**Figure 1:**
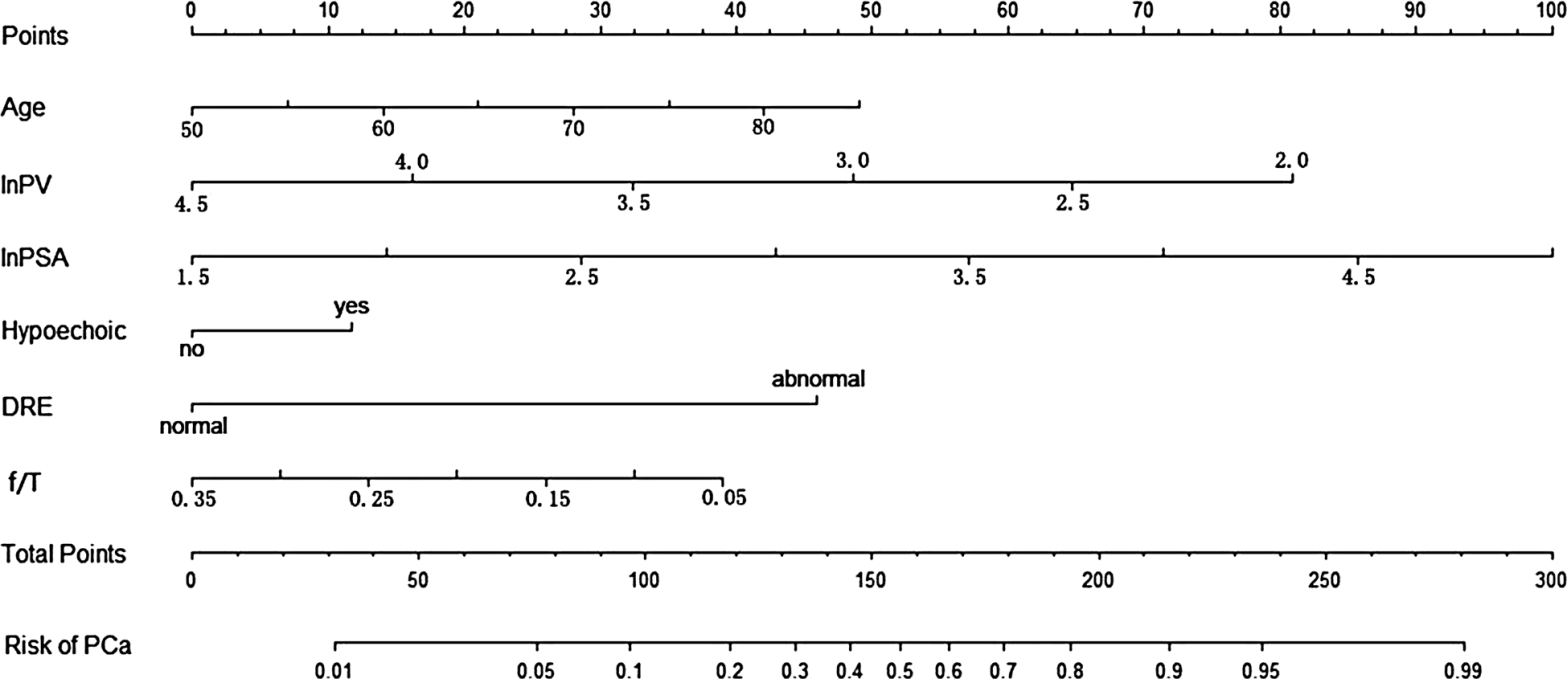
**Nomogram for predicting a positive rate.** Locate patient values on each axis, and compare to the ‘Point’ axis to determine how many points are attributed to each variable. Then locate the sum of the points for all variables on the ‘Total Points’ line to determine the individual probability of prostate cancer on the ‘risk of PCa’ line.

Variables used to build the nomogram were selected using a backward elimination scheme; only age, Ln(PSA), Ln(PV), f/t, abnormal DRE and hypoechoic lesions on TRUS were chosen for inclusion in our nomogram (Figure 1). The equation for prostate cancer probability (PCP) was derived using the logistic regression model:

$$\mathrm{PCP}=\frac{e^{‒3.577+0.054\;\left(\mathrm{Age}\right)\;‒3.714\;\left(f/t\right)\;‒1.324\left(\mathrm{Ln}\;\left(\mathrm{PV}\right)\right)\;+\;0.977\;\left(\mathrm{Ln}\;\left(\mathrm{PSA}\right)\right)\;+1.698\;\left(\mathrm{DRE}\;\mathrm{findings}\right)\;+0.458\;\left(\mathrm{hypoechoic}\right)}}{1+e^{‒3.577+0.54\;\left(\mathrm{Age}\right)\;‒3.714\;\left(f/t\right)\;‒1.324\;\left(\mathrm{Ln}\;\left(\mathrm{PV}\right)\right)\;+\;\left(\mathrm{Ln}\;\left(\mathrm{PSA}\right)\right)+\;1.698\;\left(\mathrm{DRE}\;\mathrm{findings}\right)\;+\;0.458\;\left(\mathrm{hypoechoic}\right)}}.$$

**Figure 2:**
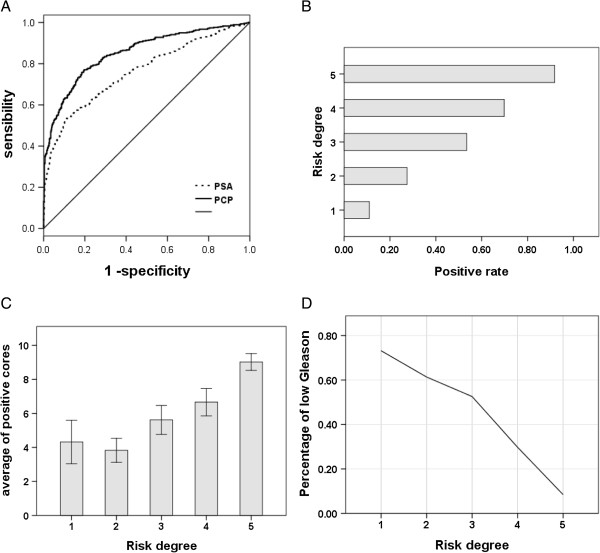
**The ROC of our nomogram and predictive information in every risk degree. (A)** The solid line represents the receiver-operating characteristic curve for the nomogram. **(B)** The strips represent the positive rate in patients at every risk level. **(C)** The strips represent the average number of positive cores taken from patients with positive results at every risk level. **(D)** The line represents the number of patients with positive results and a lower Gleason score (<7).

The PCP equation was developed to calculate patient risk level regarding the Cacoethic biopsy results. The area under the curve (AUC) of the receiver-operating characteristic curve for the nomogram is shown in Figure 2A. The AUC was increased from 0.761 for PSA alone to 0.853 for our nomogram.

In accordance with the value obtained from the PCP equation, we classified the probability of prostate cancer occurrence into five levels, and the cutoffs were at 0.2, 0.4, 0.6 and 0.8, as each demarcation point declined to a lower level. The rate of positive prostate biopsies increased in line with the risk level (Figure 2B).

In patients with positive biopsy results, the number of positive cores counted and the percentage of low Gleason scores (<7) for every risk level are shown in Figures 2C and D, respectively. Coupled with increasing risk level, the proportion of patients with lower Gleason scores decreased and there was a distinct increase in the mean number of positive cores.

Retrospective analysis of 1104 cases indicated that only a proportion of malignancies could be detected using 12-core rather than 6-core biopsy. When the PCP cutoff value reached 0.5 there was no significant difference in the detection rate between 6-core and 12-core biopsy in patients with higher PCP values (72.9% vs. 78.6%; P = 0.077); however, the difference in the detection rate in patients with lower PCP values was still significant (14.6% vs. 20.4%; P = 0.004). In patients with a PCP >0.5, 6 + 1 core biopsy was found to be more reasonable than 12 + 1-core biopsy.

From November 2012 to June 2013, a total of 238 patients were evaluated using our new nomogram; patients with a PCP cutoff value >0.5 were recommended for 6 + 1-core biopsy. The remaining patients still received systemic 12 + 1-core biopsy. The additional core was only performed in patients with hypoechoic lesions detected using TRUS. We found that 78 patients had a PCP cutoff value >0.5; 71 of these patients agreed to undergo a simplified biopsy scheme (6 + 1-core) and the other seven patients insisted on biopsy using the old scheme (12 + 1-core). Table 3 shows the comparison of data regarding the first and second stages of the study. In the second stage, we significantly reduced the number of biopsy cores (6.9 ± 2.1 vs. 13; P < 0.001) in patients with a PCP cutoff level >0.5; however, there was no significant difference in the detection rate (70.5% vs. 78.6%; P = 0.109).

**Table 3:** Comparison of the new and old biopsy schemes

	**Second stage**	**First stage**	**P**
	**n(%)**	**n(%)**	
Total			
N	238	1104	
Age	69.7 ± 7.3	68.8 ± 7.9	0.116
Biopsy cores	10.5 ± 2.8	13	<0.001
Positive rate	85(35.7)	458(41.5)	0.109
Subjects with PCP > 0.5			
N	78	384	
Age	72.2 ± 6.8	71.7 ± 6.9	0.51
Biopsy cores	6.9 ± 2.1	13	<0.001
Positive rate	55(70.5)	302(78.6)	0.138

## Discussion

Measurement of the level of PSA in serum in the elderly can help clinicians to diagnose prostate cancer at an early stage. However, other medical conditions, such as benign prostatic hypertrophy and inflammation, can elevate the PSA level in serum [14]. Because of the low specificity of the PSA level in the detection of prostate cancer, patients suspected of having this disease using PSA screening usually receive an unnecessary biopsy; this is an invasive procedure with accompanying complications [15]. The benefit of PSA screening in prolonging cancer-specific survival remains controversial [16,17]. Previous research has shown that predictive models, based on clinical, laboratory and ultrasound parameters can improve the accuracy of prostate cancer detection to varying degrees [18-20]. In the present study, Ln(PV), f/t, age, Ln(PSA), the rate of abnormal DRE findings and the rate of hypoechoic masses detected using TRUS have been taken into account for the first time. The equation used in the calculation of the PCP was derived for the diagnosis of prostate cancer. In contrast to PSA alone, use of our nomogram enlarged the AUC from 0.761 to 0.853. As shown in Figure 2B, C and D, we could evaluate the positive core rate, Gleason score and estimate the number of positive cores. In principle, a higher risk level means a higher positive rate, a higher Gleason score and more calculations regarding positive cores. As is evident in Figure 2C, the general tendency regarding the mean number of positive cores is not adequate in the first risk level; however, the difference between the first risk level and the second risk level was not significant (4.32 vs. 3.84; P = 0.473).

More recently, surgeons have used additional biopsy cores to improve the accuracy of detection of prostate cancer [9-14]. However, it has been reported in some studies that there is no significant difference between 6-core biopsy and 12-core biopsy in terms of the rate of positive biopsy cores [21,22]. According to our nomogram, when the cancer probability cutoff value reached 0.5 there was no significant difference between 6-core biopsy and 12-core biopsy at higher risk levels; however, the difference between 6-core and 12-core biopsy at lower risk levels was significant. The tumor volume ratio of the prostate may explain this. This ratio is smaller at lower risk levels, requiring the use of concentrated biopsy cores. In patients diagnosed with malignancy, the positive rate regarding the 13th core biopsy of hypoechoic masses detected using ultrasound was significantly higher than any biopsy core obtained using systemic 12-core biopsy (70.9% vs. 56.6%; P < 0.001). In summary, taking the threshold value as being a cutoff value of 0.5, 6-core biopsy was found to be adequate for patients at higher risk levels (cutoff >0.5), and 12-core biopsy was found to be adequate for the other patients. If there are hypoechoic lesions on ultrasound, performing an extra core biopsy would be helpful. This new biopsy scheme was found to reduce the number of biopsies required and did not decrease the positive core rate in the second stage of our study.

Unfortunately, our nomogram is not the first to have been developed in China. But our nomogram was based on twice the number of patients used in former nomograms involving Chinese populations (Table 4); this may have led to more reliable results. Furthermore, our nomogram has greater clinical applicability. We could roughly predict the positive core rate, ratio of positive cores and Gleason score at every risk level. Finally, with the help of our nomogram, we identified those high-risk patients in which we could reduce the number of biopsy cores by half.

**Table 4:** Comparison between our nomogram and the earlier Chinese nomogram

**Study**	**n**	**Cancer rate(%)**	**AUC for model**	**AUC for PSA**	**Increase in AUC vs. PSA alone**
Ping Tang et al.[23]	535	44.8	0.848	0.797	0.051
Our model	1104	41.5	0.853	0.761	0.092

## Conclusions

A nomogram based on data from Chinese males was developed to predict the positive core rate, ratio of positive cores and Gleason score at the various high-risk levels. A reasonable biopsy strategy based on our new nomogram was demonstrated to reduce the number of biopsy cores required in high-risk patients.

## Abbreviations

PSA: Prostate-specific antigen; PV: Prostate volume; PSAD: Prostate-specific antigen density; f/t: Free/total PSA ratio; PCP: Prostate cancer probability; ROC: Receiver operating characteristic curve; AUC: Area under the curve.

## Competing interests

The authors declare that they have no competing interests.

## Authors’ contributions

All authors participated in the study conception, design and coordination. LH, JL and CQ collected study data. GC and BL performed the data analysis. YH wrote the first draft of the paper with input of all authors. All authors read and approved the final manuscript.

## Pre-publication history

The pre-publication history for this paper can be accessed here:

http://www.biomedcentral.com/1471-2490/14/8/prepub
